# The movements made by performers in a skilled quartet: a distinctive pattern, and the function that it serves

**DOI:** 10.3389/fpsyg.2013.00841

**Published:** 2013-11-13

**Authors:** Donald Glowinski, Maurizio Mancini, Roddy Cowie, Antonio Camurri, Carlo Chiorri, Cian Doherty

**Affiliations:** ^1^NEAD, Swiss Center for Affective Sciences, University of GenevaGeneva, Switzerland; ^2^Department of Communication Computer and System Sciences, DIBRIS, CasaPaganini - InfoMus, University of GenoaGenoa, Italy; ^3^School of Psychology, Queens University of BelfastNorthern Ireland, UK; ^4^DISFOR, DISA, Department of Educational Sciences, University of GenoaGenoa, Italy

**Keywords:** joint action, music ensemble, sample entropy, expressive gesture, string quartet

## Abstract

When people perform a task as part of a joint action, their behavior is not the same as it would be if they were performing the same task alone, since it has to be adapted to facilitate shared understanding (or sometimes to prevent it). Joint performance of music offers a test bed for ecologically valid investigations of the way non-verbal behavior facilitates joint action. Here we compare the expressive movement of violinists when playing in solo and ensemble conditions. The first violinists of two string quartets (SQs), professional and student, were asked to play the same musical fragments in a solo condition and with the quartet. Synchronized multimodal recordings were created from the performances, using a specially developed software platform. Different patterns of head movement were observed. By quantifying them using an appropriate measure of entropy, we showed that head movements are more predictable in the quartet scenario. Rater evaluations showed that the change does not, as might be assumed, entail markedly reduced expression. They showed some ability to discriminate between solo and ensemble performances, but did not distinguish them in terms of emotional content or expressiveness. The data raise provocative questions about joint action in realistically complex scenarios.

## 1. Introduction

From the standpoint of a performer or a member of a live audience, music appears to be bound up with movement at multiple levels. That is a long standing observation (see, for instance, Repp, 1993), but until recently, it attracted relatively little scientific research. As noted by Palmer (2013), one factor was the technical difficulty of measuring and analyzing movement with sufficient accuracy and in sufficient quantities to address interesting questions. Alongside that were theoretical limitations inherited from research on movement in general. Richardson et al. (2010) memorably described its tendency to “place participants in experimental quarantine away from the confounds of social interaction” (p. 290). In an area where social interactions are of the essence among musicians and between audience and musicians that appears to be a substantial limitation.

With developments in both areas, a lively research field is emerging (see Sebanz et al., 2006; Knoblich et al., 2011). The work reported here contributes to it on two levels. First, it provides a sophisticated body of data, which is available to the research community, from a scenario which is a natural focus of interest: performance in a highly accomplished string quartet. Second, it describes one of the phenomena that can be observed in that data, which links to topics in the wider literature on movement. The work is set in the context of an area that is expanding on several fronts, and some of the main lines of development need to be sketched as background.

Two broad lines of development can be stated briefly. Impressionistic descriptions are increasingly being supplemented by various signal capture techniques, with motion capture as an obvious example. (It is taken as read here that the relationship is supplementing, not replacing). There is also a broad move from scenarios chosen for their simplicity to scenarios which are more complex and naturalistic. Number is part of that picture. Research with single individuals is well established, and there is an increasing body of work on duets. Quartets have been studied impressionistically (e.g., Davidson and Good, 2002; Goodman, 2002; King, 2004; Seddon and Biasutti, 2009), but work with signal capture remains limited (e.g., Glowinski et al., 2010; Keller and Appel, 2010; Moore and Chen, 2010; Papiotis et al., 2012). Orchestras, with larger numbers but a simpler communication structure, present a similar picture (see, Luck and Toiviainen, 2006; D'Ausilio et al., 2012). Another part of the picture involves the amount of expressive movement that the instrument offers. The progression there is from studies with no instrument (e.g., tapping tasks), to fixed instruments (e.g., piano), and more recently to mobile instruments (e.g., clarinet or violin). A review by Palmer (2013) provides an overview that reflects all of these trends.

A third line of development is growing awareness of the variety of functions that musicians movements may serve. Contemporary research identifies at least seven significant categories. The review by Palmer (2013) considers three options that are well recognized. Some kinds of movement provide sensory information that supports precise execution; some serve an expressive function; and some provide sensory feedback in ensembles. If we understand sensory feedback as being directly related to musical qualities (entry, tempo, loudness, etc.), then a fourth is needed. It is widely agreed that musical co-operation depends on social relationships, such as dominance, leadership, and support: see, for instance, Goodman (2002). Information related to those social issues is exchanged during performance, and movement is a key channel. For example, Davidson and Good (2002) reported nods of approval from one player to another who had executed a difficult passage well. Recently, Leman, van Noorden and their colleagues have highlighted another option, which is linked to the embodied cognition framework Leman (2007); Davis et al. (2012). They have argued that movement in response to music is part of the way in which we perceive it. For instance, we extract rhythm by establishing a pattern of movement that is synchronous with it. It is a real contribution if, for example, movement can help musicians in an ensemble to optimize their perception of the performances around them. An obvious null hypothesis is that observed movement patterns may simply be dictated by the score. Another, which is perhaps less obvious, is that they may reflect general tendencies which are of limited relevance to music. For example, Richardson et al. (2007) showed that people sitting in adjacent rocking chairs tend to rock in synchrony. We would expect similar coupling mechanisms to operate between musicians even if they served no musical function. These possibilities need to be borne in mind when more interesting hypotheses are being evaluated. It also needs to be borne in mind that in a complex performance, movement is quite likely to be serving all of the functions listed above, many of them simultaneously. That makes it an intriguing challenge to understand how performers can encode and decode the relevant information. It is also the reason why research has to engage with complex scenarios directly: simplified situations cannot show what happens when the movement channel has to support several functions at once.

A fourth line of development looks at the broad kinds of sign that serve these functions. Kurosawa and Davidson (2005) highlighted a classification due to Ekman and Friesen (1969), which has been widely adopted. It deserves detailed reading, but broadly speaking, their emphasis is on signs which are non-verbal, yet akin to language in various ways (they may translate into it, or illustrate it, or regulate it). The archetypal examples are discrete gestures, whose meaning is learned, and which are intentionally generated and consciously understood. That kind of quasi-linguistic description clearly has useful applications to music (see Davidson, 2012), but there are indications that a contrasting framework may also be useful. Goodman (2002) observed that accomplished musicians distinguish between signals which are overtly discussed and agreed, and others that are absorbed at an unconscious level (p. 158). Other descriptions hint at some of the forms that these might take. For example, Davidson (2012) describes discrete signals that can be classified along the lines proposed by Ekman and Friesen (1969); but she also describes how “the on-beats and off-beats of the clarinet to flute bounce between one another with bodily movements of sways, bobs and nods, that bring the players into a tight coordination” (p. 613). The description evokes physical phenomena, oscillation and entrainment, rather than verbal interchange. There is increasing interest in formalizing quasi-physical descriptions, from simple cases like the rocking considered by Richardson et al. (2007) to analyzing an orchestra as a set of oscillators (e.g., D'Ausilio et al., 2012).

Last but not least, the field has accumulated descriptions of individual forms of movement that feature in musical performance. The movements involved vary from instrument to instrument, but as one would expect from the conceptual distinctions above, they may relate either to expression or to social communication. Examples in the first category include swaying movements by pianists (see Davidson, 2012), and raising the bell of a clarinet (see Wanderley et al., 2005); a prominent example of the second is head-nodding (see Juslin and Sloboda, 2010).

The work described here reflects the situation which has been outlined. It contributes to research on a key scenario, the quartet, by capturing technically sophisticated data and making it available to the research community. The complexity of the situation makes it natural to collect data with a view to sustained, collaborative analysis rather than to demonstrating a single point. The analysis concentrates on a particular observation which relates to relatively understudied issues. It considers a type of movement which is naturally described in quasi-physical terms, and which seems likely to relate to musical co-ordination.

Two strategic decisions should be outlined before describing the procedure in detail.

First, the recordings centered on performances by a highly acclaimed quartet, the Quartetto di Cremona, and comparators chosen to clarify particular points. Various studies have compared expressive and inexpressive playing, with a view to identifying expressive gestures (Camurri et al., 2005a; Leman, 2007). Here, the comparison was between solo and ensemble playing, with a view to identifying movements involved in communication (Davidson, 2012). Expression cannot be ignored, but the strategy was to address it indirectly by asking for ratings of expressiveness. That provides a way of assessing whether movement patterns are linked to expressiveness or interaction. A necessary second level of comparison was to record a different quartet playing the same material. That provides a way of assessing whether observed movement patterns are idiosyncracies of the particular quartet.

Second, the movement which was studied in depth was chosen after discussion with the Quartetto di Cremona. They indicated that a subjective center of gravity, which we call the *ear* of the quartet, acted as a shared reference, to which they oriented during the performance. Since that is absent during solo performance, it appeared that relationships to it might distinguish solo and ensemble performance. The analysis which is reported in detail shows that is indeed the case. A particular reason for interest in the pattern links back to the discussion above. The changes are reminiscent of oscillating physical systems rather than discrete, language-like gestures. If that kind of effect could be documented, it would add to an area where information is relatively limited.

## 2. Experimental procedure

We recorded performances by an internationally recognized professional string quartet, the Quartetto di Cremona, and by a student quartet in the final year of study at the conservatory. Both were recorded in two conditions, playing together and as individuals. Figure 4 summarizes the various steps of the experimental procedure, involving data acquisition, pre-processing, extraction of expressive features and analysis of the way expressive features vary with social context.

### 2.1. Protocol

The SQs violinists were asked to play a famous piece: the *Allegro* of String Quartet No 14 in D minor by Schubert. Each musician played his part five times alone, and five times with the group. Five repetitions of the same 2-min length music piece without any break was considered a trade-off between the quality of the performance and the statistical requirement for replications. Musicians were instructed to play at their best, in a concert-like situation. To disentangle the possible effect of group performance on solo performance, first violinists had to perform three trials before and two trials after the group performance. The quality of each performance was assessed by the musicians through post-performance ratings (e.g., self-report level of satisfaction and expressivity).

### 2.2. Selection of the music stimuli

The piece that was used is a staple of the quartet repertoire. In order to explore features of musical structure that might affect performers' behavior, it was subdivided into five segments of about 30 s each. The score for each segment was characterized by a structure that requires particular kinds of interaction within the quartet. For example, one had a homorhythmic texture where musicians tend to play in unison, but over which first violinist emerges progressively through a subtle original motif. Another used a fugato style, where there is no leading part as such: all musicians are set at the same level by replicating the musical subject over the different instruments.

### 2.3. Setup

**Figure 1:**
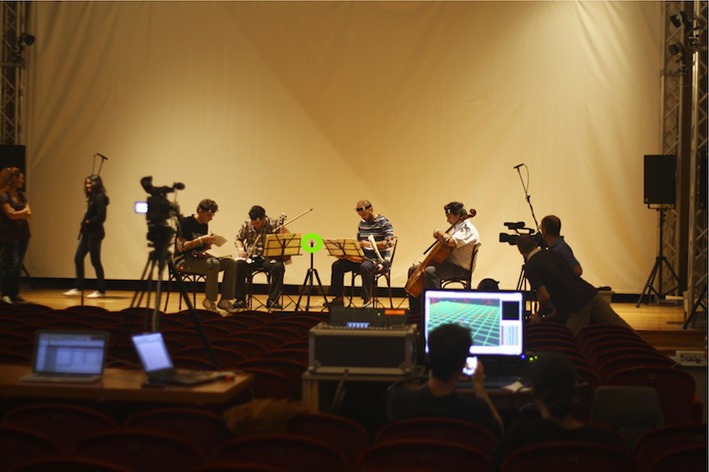
**The multimodal setup for the experiment, showing motion capture, videocameras, and environmental microphones**. Each musician wears markers for motion capture. Piezoelectric microphones are in the body of the instrument. Note the position of the SQ ear represented by the optical reflector placed on the tripod situated in the center of the quartet, equidistant from the four musicians.

The experiment took place in a 250-seat auditorium. It provided an environment similar to a concert hall, but equipped to allow experiments in an ecologically valid setting (see Figure 1). The setup incorporated multiple capture modalities: motion capture, using the Qualisys system (www.qualisys.com); video cameras; environmental stereo microphones; and piezoelectric microphones attached to the body of each instrument. Inputs from all the modalities were synchronized using real-time applications developed within the EyesWeb XMI software platform (Camurri et al., 2005b).

### 2.4. Data

This paper focuses on one particular component of the recordings, the time series data of the musician's head distance to the so-called “ear” of the SQ.

**Figure 2:**
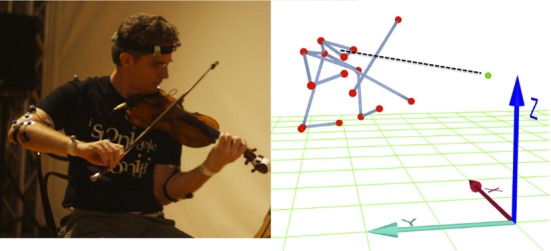
**Picture and Motion Capture (MoCap) data of the first violinist of Quartetto di Cremona**. The dashed line represents the main dependent variable, that is, the distance between the musician's head center of gravity (COG) and the ear, the subjective center of the string quartet.

**Figure 3:**
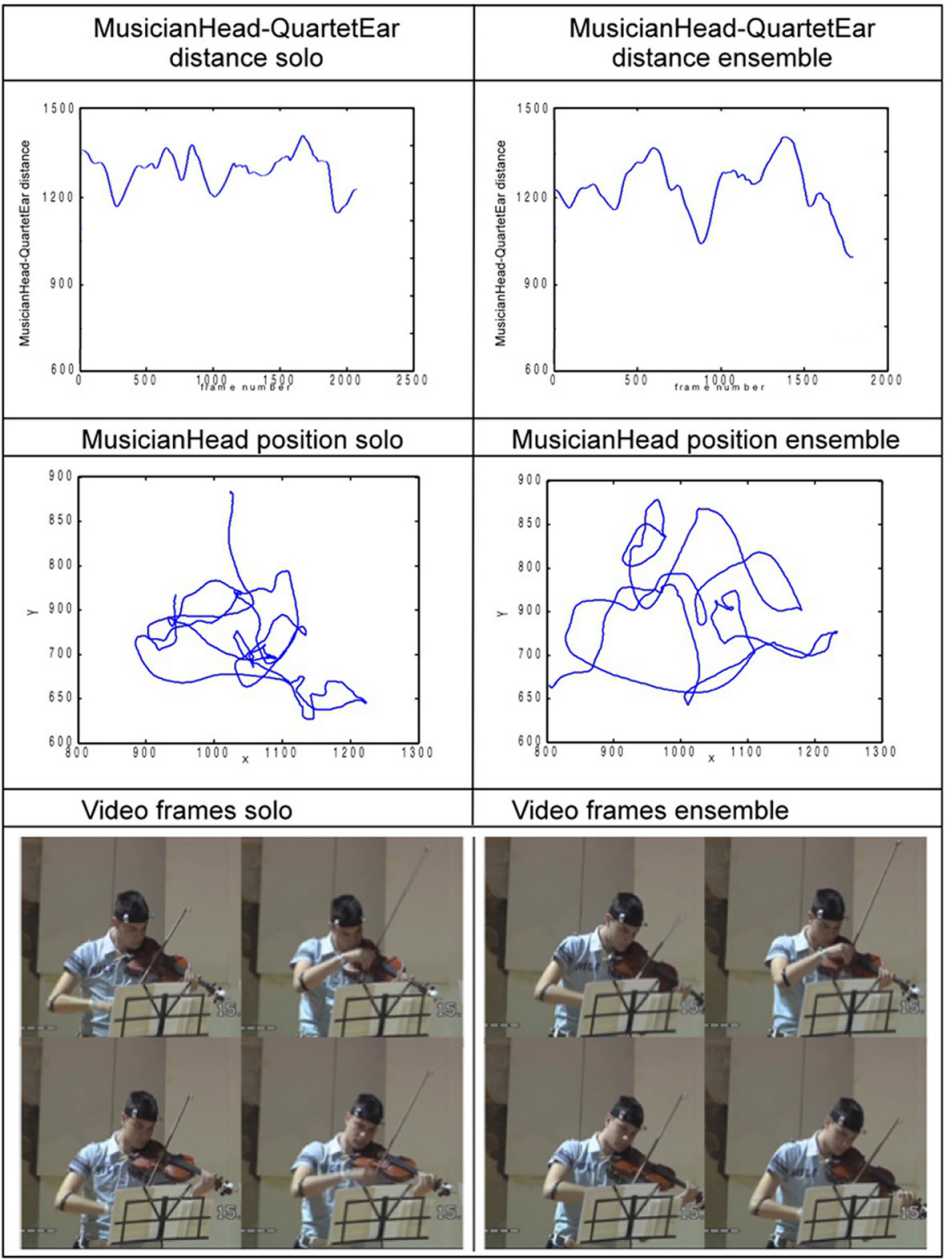
**Visualization of head data related to the first violinist of the student quartet in Solo and Ensemble conditions for a typical (brief) passage**. The **upper panels** show distance between the head center of gravity (COG) and the ear; the **middle panels** show the corresponding path of the COG as viewed from above, and the **lower panels** show frames from the excerpts.

Head movements are a natural choice because they are known to play a central role in non-verbal communication in general (e.g., Glowinski et al., 2011) and in music in particular (e.g., Davidson, 2005; Castellano et al., 2007; Dahl et al., 2009). Known functions range from providing explicit markers (used to achieve synchrony at the beginning of a passage) to conveying emotional states (either as a matter of self-expression or to communicate relevant feelings of appreciation or reassurance to others), see Camurri et al. (2005b). The specific measure that we extracted is the distance between the head and the subjective center of the SQ, the ear. For a musician's movements to impact upon the ensemble, the other performers need to be able to recognize that the behavior is addressed to them. The area surrounding the ear of the SQ has a special meaning for the musicians, which is bound up with their sense of the quartet as a unit. It makes sense that movements relative to that focus should have a particular significance for co-ordination. Note that before the recording, the position of the ear was discussed with the musicians and physically implemented using an optical reflector set on a tripod (see Figure 1). On that basis, we analyzed how the distance from each musician's head to the ear varied during performance. For the head, a center of gravity (COG) was computed starting from the three markers placed on the musician's head, two on the front and one in the back (see Figure 2). Euclidean distance between the head COG and the String Quartet's ear was then computed for each frame. Following the recommendations in Ramdani et al. (2009), analysis was conducted on the increment of the head COG/Quartet's ear distance time series (see Figure 3).

### 2.5. Method

The regularity of head movement was analyzed by considering Sample Entropy (SampEn). SampEn is a non-linear technique that was initially developed to quantify behavior regularity by Richman and Moorman (2000) and improved by Govindan et al. (2007). The main difference between this measure and traditional time and frequency domain techniques (e.g., spectral analysis) is that SampEn considers the recent movement history. For example, suppose that a player keeps time with a regular rhythm by swinging her head forward and backward in a periodic way, and then she suddenly increases her head excursion at the beginning of a more animated musical phrase. The corresponding SampEn distinguishes this sudden change in motion. A traditional entropy approach would consider each frame as a separate event and compute an average value of entropy for each, ignoring the history of the input signal. SampEn has been applied to a variety of physiological data [heart rate, EMG, see Seely and Macklem (2004) for a review]. Most recent applications deal with behavioral data (e.g., investigating postural control mechanisms; Ramdani et al., 2009) and some specifically address affective and social dynamics (Glowinski et al., 2010). As usual with entropy measures, higher values of SampEn indicate higher disorder, and smaller values indicate greater regularity. The computation of sample entropy is described in the next section.

#### 2.5.1. SampEn algorithm

Given a standardized one-dimensional discrete time series of length *N*, *X* = {*x*_1_, …, *x*_*i*_ …, *x*_*N*_}:

1. construct vectors of length m [similarly to the time delay embedding procedure in Takens (1980)],
  (1) $$u_{i}(m)=\{x_{i},\;\ldots ,\;x_{i\;+\;m\;-\;1}\},\;1\leq i\leq N-m$$
2. compute the correlation sum *U*^*m*^_*i*_(*r*) to estimate similar subsequences (or *template vectors*) of length m within the time series:
  (2) $$U_{i}^{m}(r)=\frac{1}{\left(N-m-1\right)}\sum_{i=1,\;i\neq j}^{N-m}\Theta (r\;-\;∥u_{i}(m)-u_{j}(m){∥}_{\infty })$$
  where *u*_*i*_(*m*) and *u*_*j*_(*m*) are the template vectors of length *m* formed from the standardized time series, at time *i* and *j*, respectively, *N* is the number of samples in the time series, *r* is the tolerance (or *radius*), Θ is the Heaviside function, and ‖ ‖_∞_ is the maximum norm defined by ‖ *u*_*i*_(*m*) − *u*_*j*_(*m*) ‖_∞_) = max_0 ≤ *k* ≤ *m* − 1_ | *x*_*j* + *k*_ − *x*_*i* + *k*_ |.
3. calculate the average of *U*^*m*^_*i*_, i.e., the probability that two vectors will match in the *m*-dimensional reconstructed state space
  (3) $$U^{m}(r)=\frac{1}{\left(N-m\right)}\sum_{i=1}^{N-m}U_{i}^{m}(r)$$
4. set *m* = *m* + 1 and repeat steps 1–4
5. calculate the sample entropy of *X*_*n*_
  (4) $$\text{SampEn}(X_{n},\;m,\;r)=-ln\frac{U^{m\;+\;1}(r)}{U^{m}(r)}$$

SampEn computes the negative natural logarithm of the conditional probability that subsequences similar for *m* points in the time series remain similar (as defined by Equation 3) when one more point (*m* + 1) is added to those sequences. Hence, small values of SampEn indicate regularity. Following Ramdani et al. (2009), parameters of SampEn were set to *m* = 3 and *r* (tolerance) = 0.20.

### 2.6. Analysis of the score

It could be argued that the behavior irregularity observed during the experiment might be a product of the complexity of the musical task faced by the musician (e.g., more notes with higher intervals to play may result in more complex movement to execute). To disentangle the effects of structural features of the music as distinct from the interpersonal dynamics within the group, an analysis of the complexity of the musical score was carried out. For each of the five musical segments played in the experiment, the individual musicians' parts were evaluated using the expectancy-based model of melodic complexity (Eerola and North, 2000). This results in a unique index for each musician's part, based on the variety of intervals, the rhythmic and melodic densities encountered in each musician's part, a unique index is given. Friedman's non-parametric repeated measures analysis of variance was conducted to compare the melodic complexity index between musicians, over the five music segments.

### 2.7. Perceptual study

A perceptual experiment studied (1) observers' ability to recognize the social context (solo vs. ensemble) of the musical performance based on non-verbal behavioral information (Juslin and Laukka, 2003), and (2) their evaluation of its expressiveness. Forty samples were selected for perceptual analysis from the full set of audio–video recordings of the first professional and student violinist's performance. The selection of the recordings was based on the annotations made by the musician after each of his performances. We ensured that a broad range of expressive performance qualities could be represented in our sample recordings by considering the annotation given by the musician (e.g., worst and best interpretations). Participants completed a short socio-demographical schedule asking to report gender and age and rate their self-reported level of empathy on a five-point scale. Audio–video recordings were displayed via a flat screen (17) and headphones (Sennheiser). In a random half of the trials the musician was playing solo, in the other half was playing with other musicians of the string quartet. Participants had (1) to report whether they thought the performance was solo or ensemble and (2) to evaluate its level of expressivity on a five-point scale. Two groups of participants were selected: non-expert and music expert (with a minimum of 6 years of music practice in music school). Forty-eight participants (17 non-experts and 31 experts) took part to the study. Twenty-five saw recordings of a student violinist, 23 of a professional player.

## 3. Evaluation

### 3.1. Perceptual and score-based analysis

Comparisons covered two issues: whether raters could identify the context in which performances occurred (solo or in the ensemble), and how expressive they judged the performances to be. With regard to context, a Fisher's exact test showed that there was a significant (*p* < 0.05) association of Condition (Solo vs. Ensemble) with the Perceived Condition (Solo vs. Ensemble). It means that correct identifications were more likely than chance. A more sophisticated analysis based on Signal Detection Theory used the area under the curve (AUC) as a measure of the accuracy with which participants could distinguish between ensemble and solo performances. Factorial analysis of covariance models were specified including age, gender, self-reported empathy level as covariates and type of violinist (professional vs. student) and expertise (musician vs. non-musician) as factors. The main effect of type of violinist (*p* = 0.020) was the only statistically significant effect, with higher AUCs for the performances by professional violinists [AUC mean values with their confidence intervals are respectively 0.69 (0.63–0.75) vs. 0.60 (0.54–0.65)]. Both are statistically different from 0.50, which is the rate expected by chance. Expertise emerges as an issue in both analyses. The differences are more perceptible to expert raters (though not significantly so), and raters are significantly better able to distinguish between solo and ensemble performances by expert musicians.

With regard to expressivity, there were no significant main effects. There was a curious interaction in which experts rated the performance more expressive if they believed (rightly or wrongly) that the performance was in ensemble, whereas non-expert raters showed the opposite pattern. That seems to reflect different *a priori* beliefs, and underlines the fact that actual differences in the performances were slight.

### 3.2. Movement data analysis

The SampEn measure was calculated for three musicians. They were the first and second violins from the Cremona quartet (including different roles provides a way to check whether effects are specific to role); and the first violin from the student quartet (providing a way to check whether effects might reflect idiosyncratic habits in a particular group). Considering the unbalanced repeated measures design (six observations for solo condition and five for the quartet condition), *sphericity* could not be assumed. Corrections due to Greenhouse–Geisser and Hyunh–Feldt, could be applied but they are not optimal ways of handling correlated data and unequal variance. Instead a linear mixed model (LMM) approach was chosen to compare musicians' SampEn values across conditions (McLean et al., 1991). To control the inflation of type I error probability due to multiple comparisons, the Bonferroni correction was applied to adjust the *p*-values required for statistical significance. Applied on the full set of 165 samples (55 for each musician), LMM identified significant main effects of Condition (Solo > Ensemble, *p* < 0.001), Musician (Professional violinist > Student violinist, *p* < 0.001), and Music Segment (*p* < 0.001). A number of significant interaction effects have also been identified: Condition x Segment interaction (*p* = 0.042) and Condition x Musician x Segment (*p* < 0.001). Bonferroni-corrected *post-hoc* analyses were performed to assess specific difference among the Conditions, Segments, and the Condition × Music Segment interaction effects. We review in the following the main effect of *Condition* and the related *Condition* × *Music Segment* interaction effect.

***3.2.0.1. Main effect of Condition***. Results showed that the experimental condition had a significant main effect on SampEn values: considering the musicians altogether, for all segments, over all takes, SampEn values in the Solo Condition were significantly higher than in Ensemble Condition [*F*_(1, 135)_ = 119.984, *p* < 0.001], see Figure 5.

***3.2.0.2. Condition x Music Segment interactions***. *Post-hoc* analysis of the Condition x Music Segment interaction revealed that SampEn values of musicians are significantly higher in Solo Condition vs. Ensemble for all music segments (*p* < 0.001), see Figure 6. Within Solo condition, music segments 1 and 5 are statistically lower than other music segments (*p* < 0.001) and within Ensemble condition, music segments 1 and 5 are, respectively statistically (*p* < 0.001) and marginally significant (*p* = 0.07).

## 4. Discussion

Figure 3 illustrates an intriguing difference in the quality of movement observed in solo and ensemble playing. The SampEn measure captures it in a way that lends itself to statistical analyses, and makes it possible to show that there is a stable phenomenon to be considered. A characteristic which is immediately interesting is that it involves sustained change in patterns of oscillation. Features of that kind are not unique, but they are less often described than discrete, broadly language-like gestures. However, the numerical data alone leave a great many questions unanswered, and in particular questions about the function if any that the change in quality of movement serves. The richness of the material means that many of them can be answered at least tentatively by combining sources of evidence and using the conceptual resources outlined in the introduction.

The most immediate question is whether the observed differences are statistical accidents. That has two levels. The first level is whether it is due to variability across repetitions. The statistical analyses used show that is highly unlikely. The second is whether it is an idiosyncratic behavior associated with the particular violinists who were considered. Given the small number of individuals involved, that cannot be ruled out definitively. However, given that the pattern occurs across individuals with different roles and in different quartets, it would seem unlikely. Related to those is the possibility that the effects arose because participants intuited what the experimenter expected, and obliged. Participants certainly may have intuited that the experimenter expected there to be differences between solo and ensemble performance, and that would be a problem if the claim were simply that the two are different. However, experimenter effects cannot explain why differences took the particular form of change in entropy, not least because the experimenters had not anticipated in advance that they might.

A more interesting question is whether the behavior relates to communicating a specific socially defined role (King, 2006). In fact, the effect was noticed in the first violin of the Quartetto di Cremona, and the initial hypothesis was that the simplification facilitated leadership (Glowinski et al., 2012). However, that can be ruled out because the effect also occurs in the second violin.

Those arguments leave the possibility that the change is associated with expression. The literature does not immediately resolve the issue. Studies of movable instruments suggest that expression is associated with microgestures (Kim et al., 2010). Introducing microgestures would be expected to increase entropy. The difference between musicians is in the direction that would be expected if high entropy were associated with expressive playing (entropy is highest in the Cremona first violin, and lowest in the student). Hence the change from solo to ensemble playing could signal that musicians sacrificed expression to cohesion when they were playing in the ensemble. In fact, though, the perceptual studies show that expressiveness does not change substantially. Hence that kind of explanation seems unlikely.

Another option which remains is that the phenomenon involves automatic social alignment, like the alignment that occurs between people in rocking chairs, and has no musical significance (Richardson et al., 2007). Again, the evidence has features which make that unlikely. In particular, the interaction shown in Figure 6 shows that effect is specific to certain musical pieces. It would seem, then, that it is in some way related to achieving certain kinds of performance. That point is expanded below.

Research by Goebl and Palmer (2009) suggests a related interpretation. They used synchronous head movements as a measure of rapport. Lowered entropy could reflect adjustments that reflected rapport, but had no function of their own. However, on that account, the obvious expectation would be that the effect would be greatest for piece 1, where the musicians are aligned in rhythm, and often in melody. In fact, as Figure 6 shows, it is the passage where the effect is smallest.

Ruling out options is not an exciting activity. Nevertheless, the fact that music has many potential functions in an ensemble makes it an important exercise: the interpretation that first comes to mind may well not be the right one. In this case, eliminating the unlikely leaves at least two options, which are not wholly separate. One is that the *simplification of movement has a communicative function*: it supports the unconscious exchange of information between musicians, along the lines that Goodman describes (Goodman, 2002). The other is that it contributes to an *embodied perception strategy* (Davis et al., 2012). Roughly speaking, regular movement provides a kinaesthetically defined framework within which musicians can locate auditory events (Keller and Appel, 2010). That goes further than existing arguments in the area, notably the argument by Leman et al. (2009) that matching body movements mediates perception of musical attributes, but the extension is a reasonably natural one.

Both explanations are compatible with the contrasts between pieces. Piece 1, where the difference is small, is homorhythmic, and often in unison; hence, there are multiple confirmations of the rhythmic framework. That changes in the later segments, where the style shifts first to confrontation, then to contrasting melody and accompaniment, then to a temporally offset fugal style, and to a contrapuntal style. Hence it is broadly reasonable to propose that simplified and synchronized movement functions to establish a shared rhythmic framework when auditory input from other performers is rhythmically challenging.

The two theoretical interpretations that have been outlined, involving inter-performer communication and embodied perception, are not incompatible. In many ways the most natural assumption is that both are at work. Rhythmic behavior could well be an input both to the performer who generates it, and to the performer who perceives it. Given evidence that there are automatic processes which tend to synchronize the rhythmic movements of people close to each other, they may also be recruited to the process when the need arises (Richardson et al., 2007; Goebl and Palmer, 2009; Vesper et al., 2011).

What has been sketched here is a hypothesis. It is consistent with a complex body of evidence, with pre-existing theory, and with experience as a performer. More positive confirmation is clearly desirable, and the richness of the data mean that several possibilities can be envisaged. SampEn is a powerful summary, which allows statistical analysis: but given the results of the analyses, it becomes useful to look more closely at the movement patterns that underlie it. If those patterns are a way of coping with rhythmic demands, then one would expect to find more direct evidence of difficulty co-ordinating rhythm in the areas where they are present. If rhythmic behavior is an input to other performers, then one would expect looking behavior to change when it was being used. All of these involve substantial analyses, but they become worth undertaking if there is a credible and interesting hypothesis to investigate.

## 5. Future work and conclusion

It is already clear that musicians playing together use many kinds of movement for many kinds of purposes, as witness, for example, the descriptions brought together by Schutz (2008) and Davidson (2012). Nevertheless, it seems likely that there is still a great deal more to be discovered about the way that movement features in music. With the increasing use of signal capture techniques, one of the areas that are opened up involves movement patterns that are more akin to physical oscillation and resonance than to discrete linguistic gestures. The basic finding here, that at least some such patterns do change between solo and ensemble performance, encourages further research into that general area; and the recordings provide a resource that is valuable to it.

It is probably not an accident that attributing functions to that kind of change is a delicate and uncertain exercise. Function can often be equated with the goal that an action is intended to achieve, but it seems quite likely that intention in the classical sense has very little to do with this kind of change in the quality of movement. It also seems very likely that such changes often involve many to one mapping: multiple internal factors influence the external signal, which is a particular kind of movement.

**Figure 4:**
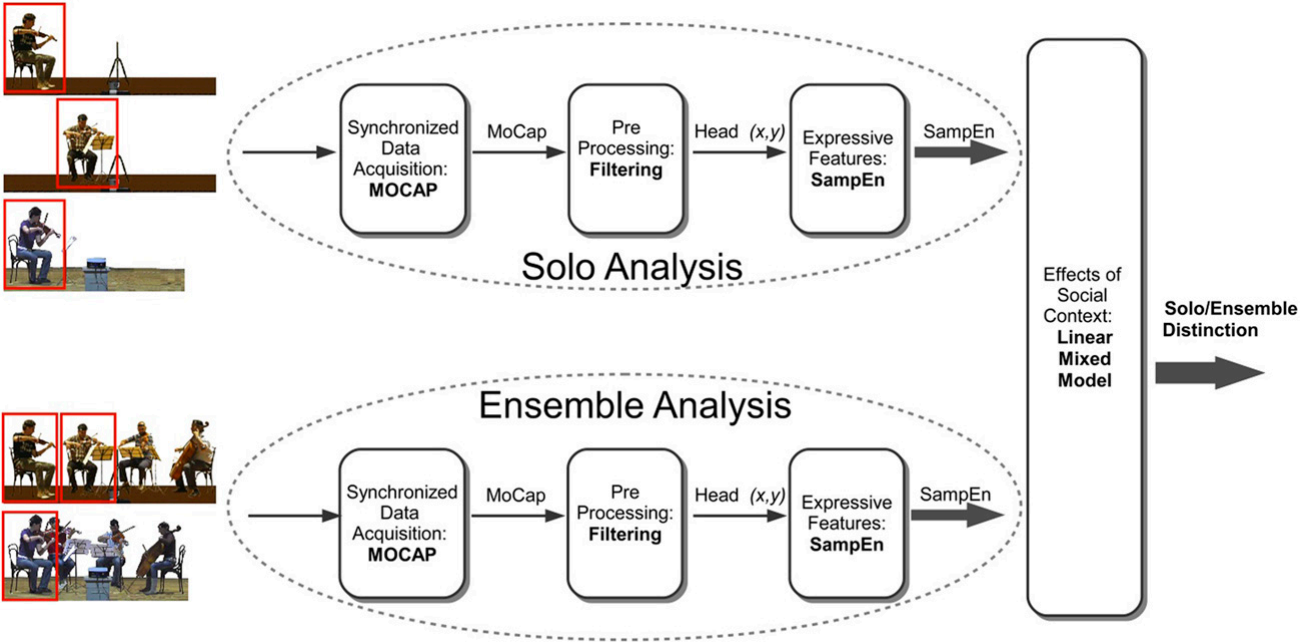
**Visualization of the data processing and analysis steps in evaluation of the difference between solo and ensemble performance**. Research on complex patterns of behavior requires attention to data acquisition, preprocessing, extraction of expressive features, and analysis of the way expressive features vary with social context. In the present study, we extracted expressive features of violinists playing in a solo and in a string quartet, respectively. Violinists' head movements were obtained by the Qualisys motion capture system, and we analyzed the regularity of head movements using a measure of entropy (SampEn).

If the phenomena are as complex as that, and as difficult to capture in words, then there are implications for the methods that are relevant to studying them. In general, building extended models seems more likely to be productive than attempting to confirm or refute isolated hypotheses. That is only possible if empirical research accumulates suitably large and coherent bodies of data. The approach to data collection that is adopted here reflects that judgment. The scheme of analysis shown in Figure 4 embodies a framework for what is probably the most easily developed kind of computational analysis, that is, one which aims to identify different kinds of performance from sensor inputs.

**Figure 5:**
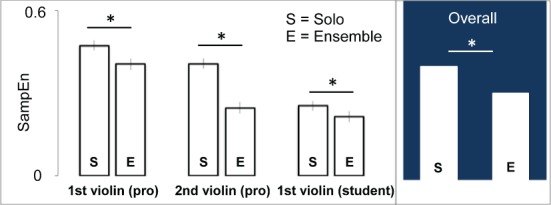
**Main effect of condition *Solo* vs. *Ensemble***. The histogram represents the amount of SampEn for Solo (S) and Ensemble (E) performances, separately for professional and student violinists. Asterisks denote significant differences. In blue background, the average over the two conditions for both musicians taken altogether.

**Figure 6:**
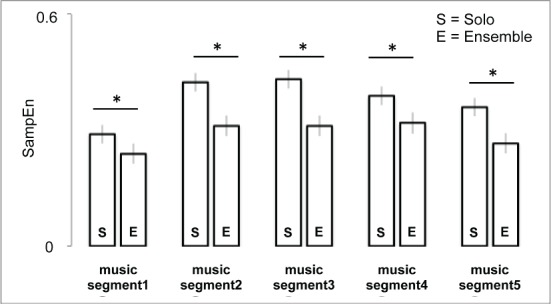
**Condition × music segment interaction effect**. The histograms represent the amount of SampEn separately for each of the five music segments, for both conditions Solo (S) and Ensemble (E). In each sub-figure the lower bars represent significant comparisons. Asterisks denote significant differences.

One reaction, which is wholly understandable, is to avoid such unaccommodating phenomena. However, exactly the opposite response also makes sense. One of the reasons for studying movement in ensemble performance is precisely that it exposes features of the way humans interact that we otherwise tend to overlook, not least because they press us to look beyond everyday ways of thinking about human beings. That, at root, is the motive behind this research. It is because they are provocative that the phenomena are interesting.

### Conflict of interest statement

The authors declare that the research was conducted in the absence of any commercial or financial relationships that could be construed as a potential conflict of interest.
